# Antileishmanial activity of a mixture of *Tridax procumbens* and *Allium sativum* in mice

**DOI:** 10.1051/parasite/2014016

**Published:** 2014-04-10

**Authors:** Rubi Gamboa-Leon, Marina Vera-Ku, Sergio R. Peraza-Sanchez, Carlos Ku-Chulim, Aurelio Horta-Baas, Miguel Rosado-Vallado

**Affiliations:** 1 Laboratorio de Parasitología, Centro de Investigaciones Regionales “Dr. Hideyo Noguchi”, Universidad Autónoma de Yucatán (CIR-UADY) Avenida Itzáes # 490 × Calle 59 Colonia Centro 97000 Mérida, Yucatán México; 2 Centro de Investigación Científica de Yucatán (CICY) Calle 43 No. 130, Col. Chuburná de Hidalgo 97200 Mérida, Yucatán México

**Keywords:** Leishmanicidal activity, *Tridax procumbens*, *Allium sativum*, Acute oral toxicity, Phytomedicine

## Abstract

We tested a mixture of *Tridax procumbens*, known for its direct action against *Leishmania mexicana,* and *Allium sativum,* known for its immunomodulatory effect, as an alternative to treat cutaneous leishmaniasis. Acute oral toxicity was tested with the Up-and-Down Procedure (UDP) using a group of healthy mice administered with either *T. procumbens* or *A. sativum* extracts and compared with a control group. Liver injury and other parameters of toxicity were determined in mice at day 14. The in vivo assay was performed with mice infected with *L. mexicana* promastigotes and treated with either a mixture of *T. procumbens* and *A. sativum* or each extract separately. The thickness of the mice’s footpads was measured weekly. After the 12-week period of infection, blood samples were obtained by cardiac puncture to determine the total IgG, IgG1 and IgG2a immunoglobulins by a noncommercial indirect ELISA. We showed that the mixture of *T. procumbens* and *A. sativum* extracts was better at controlling *L. mexicana* infection while not being toxic when tested in the acute oral toxicity assay in mice. An increase in the ratio of IgG2a/IgG1 indicated a tendency to raise a Th1-type immune response in mice treated with the mixture. The mixture of *T. procumbens* and *A. sativum* extracts is a promising natural treatment for cutaneous leishmaniasis and its healing effects make it a good candidate for a possible new phytomedicine.

## Abstract (fr)

Nous avons testé un mélange de *Tridax procumbens*, dont l’action directe contre le parasite *Leishmania mexicana* est connue, et d’*Allium sativum*, dont l’effet immunomodulateur est connu, comme une alternative pour traiter la leishmaniose cutanée. La toxicité orale aiguë a été testée avec la procédure Up and Down (UDP) sur un groupe de souris saines auxquelles ont été administrés soit des extraits de *T. procumbens*, soit des extraits de *A. sativum*, comparé à un groupe de contrôle. Les lésions hépatiques et d’autres paramètres de toxicité ont été déterminés au 14ème jour. L’essai in vivo a été réalisé avec des souris infectées avec *L. mexicana* et traitées soit avec un mélange de *T. procumbens* et *A. sativum*, soit avec les 2 extraits séparés. L’épaisseur des coussinets plantaires des souris a été mesurée une fois par semaine. Douze semaines après l’infection, un échantillon de sang a été prélevé par ponction cardiaque et les quantités totales d’immunoglobulines IgG, IgG1, et IgG2 ont été déterminées par ELISA indirect non commercial. Nous avons pu montrer que le mélange d’extraits de *T. procumbens* et *A. sativum* contrôlait mieux l’infection tout en n’étant pas toxique lors de l’essai de toxicité orale aiguë chez la souris. L’augmentation du ratio IgG2a/IgG1 a permis d’indiquer une tendance à augmenter une réponse immunitaire de type Th1 dans les souris traitées avec ce mélange. Le mélange des extraits de *T. procumbens* et *A. sativum* est un traitement naturel prometteur pour la leishmaniose cutanée et ses effets curatifs en font un bon candidat pour un possible nouveau phytomédicament.

## Introduction

The disease caused by infection with *Leishmania* parasites has an important impact worldwide, with up to 200 million new cases per year [3]. In Mexico, the Yucatan peninsula is an endemic area for *Leishmania mexicana*, which causes localized cutaneous lesions, known popularly as “chiclero’s ulcer”, with an incidence of 5/1,000 inhabitants [4, 18], although it can also induce more severe forms of the disease such as the diffuse, mucocutaneous, or visceral forms [8]. Chemotherapy with antimonials is the first choice to treat leishmaniasis; however, the treatment is prolonged, expensive and not very effective, and may have severe side effects. The second-line drugs, such as amphotericin B and pentamidine, may even be more toxic. Besides the problems already mentioned, [28] reported increasing failures of miltefosine, currently used to treat visceral leishmaniasis, some of the possible causes being parasite resistance, discontinuity of treatment, and reinfection.

Currently, there is an urgent need for new leishmanicidal drugs and it is known that medicinal plants are an important source of new molecules with pharmacological activities [10, 24, 29]; several plants have been used as treatment for “chiclero’s ulcer” in Mayan traditional medicine. A number of them has been screened looking for in vitro leishmanicidal activity [15]. *Tridax procumbens* L. (Asteraceae), also known as bull’s herb in Guatemala and *t’ulum* (Maya language) in Yucatan, Mexico, was one of the most active [25, 26]. The whole plant of *T. procumbens* is used by the population in Guatemala for topical applications to treat chronic ulcers caused by leishmaniasis [7]. Based on its activity, *T. procumbens* was chosen for further chemical, in vitro and in vivo studies. Methanol extract had a damaging effect on *L. mexicana* by means of a mechanism of action independent of the production of nitric oxide (NO) in macrophages [19, 20], which suggests that its activity is not cell-mediated but toxic to the parasite. Accordingly, *T. procumbens* was selected for further in vivo studies.

*Allium sativum* (garlic) is another species that has shown activity against infection with *L. mexicana*, this time through an immunomodulatory effect that induces a Th1-type response, INF-*γ* increase, and stimulation of nitric oxide (NO) production in macrophages, which prevent the progress of the infection [12]. These results are consistent with other studies, in which *A. sativum* extract also showed an inhibitory effect against infection with other *Leishmania* species [16, 32].

Since our aim is related to the discovery of potential new pharmaceutical treatments from natural sources, we considered it imperative to perform an acute oral toxicity assay to confirm the antiprotozoal activity as opposed to general toxicity and to improve the selection process for promising extracts [31]. We have observed that a large number of reports on the in vitro antiprotozoal activities of plant extracts without reference to toxicity have been published elsewhere [5], thus it is not always clear if the observed activity could be related to a general toxicity. On this basis, in the present study we tested the acute oral toxicity of *T. procumbens* methanol and *A. sativum* aqueous extracts, before testing the in vivo activity of both, separated or combined, against a *L. mexicana* infection. On the other hand, we were also interested in knowing the kind of immune humoral response (total IgGs) and the IgG2a/IgG1 ratio (Th1-type response) of all treated mice whose *L. mexicana* lesions were controlled.

## Materials and methods

### Plant collection and extraction

1.5 kg of *Tridax procumbens* L. (Asteraceae) whole plant were collected in Merida, Yucatan, Mexico, in February 2004; a voucher specimen was authenticated by F. May-Pat and deposited at the herbarium of the CICY under the code number FMay-1955. The whole plant was first dried at room temperature and then in a desiccation stove at 50 °C, powdered, and then submitted to extraction with methanol (MeOH). The MeOH was evaporated to dryness *in vacuo* and the yield of the resultant crude extract was 150 g (yielding 10%). 1.5 kg of *Allium sativum* L. (Amaryllidaceae) (garlic) bulbs were peeled and dried in a desiccation stove at 100 °C, and after 48 h they were recovered at constant weight. The dried bulbs were homogenized with a blender in 1 L of water, filtered through Whatman No. 1 filter paper, and centrifuged. The filtrate was lyophilized, dosed and resuspended for treatment.

### Parasites

Promastigotes of *L. mexicana* (strain Hd18-(MHET/MX/97/Hd18) were cultured in Tc 199 medium (Gibco, Invitrogen), supplemented with 10% fetal calf serum (Life Technologies), penicillin (100 U/mL), and streptomycin (100 g/mL). The parasites were harvested in the stationary phase after 8–10 days of culture, centrifuged (1000 g × 5 min), washed twice with Tc 199 medium, counted, and used to inoculate mice.

### Mice

Either female BALB/c or CD-1 mice (6–8 weeks old) were fed with standard chow and water ad libitum and kept under standard temperature (26 °C and 60% humidity conditions) and a natural light-darkness cycle. All in vivo experiments with mice were performed according to the established bioethical standards of the CIR-UADY Bioethics in Research Committee.

### Acute oral toxicity assay

Acute oral toxicity was tested using the Up-and-Down Procedure (UDP) recommended by the United States Environmental Protection Agency (EPA) and adopted by the Organization for Economic Cooperation and Development (OECD). This procedure significantly reduced the number of animals used in comparison with the traditional LD_50_ test [11].

Sixteen healthy, eight-week-old BALB/c mice were used to perform this test; six mice were used as a control group (saline solution) and ten were used as experimental groups (*T. procumbens* and *A. sativum* extracts). Animals were randomly selected and marked to allow individual identification. They were acclimatized and individually kept in their cages for at least five days, and fasted 24 h prior to dosing. Food and water were withheld for 3–4 h after dosing. First, two mice were dosed at 2000 mg/kg of body weight with either *T. procumbens* or *A. sativum* extracts using distilled water as a vehicle; as the mice survived, another eight were administered with the same dose of either *T. procumbens* or *A. sativum*. The control group was dosed only with the vehicle. Each mouse of the experimental groups was carefully observed in the first 3 h to look for toxicity parameters, such as tremors, convulsions, breathing changes, piloerection, loss of balance, hyperactivity, and lethargy, then animals were housed for 14 days counted from the dosage day. The parameters observed during these days were body weight, amount of food consumed, and any behavior that could be a sign of toxicity [6]. At day 14, the mice were sedated and blood was taken by heart puncture to determine liver injury. For toxicity assays in mice models, it is a good parameter to determine the increase in glutamate transaminase activities (GOT and GPT), which is a sign of liver injury, thus we determined the aspartate aminotransferase (ASAT) and alanine aminotransferase (ALAT) using the IFCC Mod. LiquiUV Test. The mice were killed to remove the liver and spleen and evaluate their appearance and weight [22].

### *In vivo* assay of *T. procumbens* and *A. sativum* extracts and their mixture

All mice were infected subcutaneously into the left hind footpad with 10^6^ logarithmic phase promastigotes of *L. mexicana* to a final volume of 50 μL of physiological saline solution. The contralateral right footpad received an identical volume of saline solution without parasites as an internal control.

The in vivo assay was performed using four groups of six mice: (1) the control (saline solution), (2) treatment with *T. procumbens*, (3) treatment with *A. sativum*, and (4) treatment with a mixture of *T. procumbens* and *A. sativum*. The treatments with *T. procumbens* and *A. sativum* extracts were started two weeks after infection. The first group was used as infection control receiving saline solution injections with the same schedule as the groups treated with extracts. The other three groups were treated daily, for two weeks, by intraperitoneal (i.p.) injections of 20 mg/kg of either *T. procumbens* methanol extract or *A. sativum* aqueous extract, or 40 mg/kg of a mixture of *T. procumbens* and *A. sativum* extracts (1:1).

The thickness of infected and uninfected footpads was measured weekly with a vernier caliper, for 12 weeks, and the difference between the two measurements was considered as the size of the lesion. The mice were also regularly examined to detect cutaneous ulcers and secondary lesions. After the 12-week period of the experiment, the mice were anesthetized and a blood sample was obtained by cardiac puncture, and they were euthanized immediately after.

### Immunoglobulin detection

The total IgG, IgG1 and IgG2a immunoglobulins were measured by a noncommercial indirect ELISA, using as an antigen a whole parasite lysate and goat anti-mouse immunoglobulin G (IgG)-horseradish peroxidase conjugate (Sigma) at 1:4000 or goat anti-mouse IgG1- or IgG2a-horseradish peroxidase-conjugated antibodies (Southern Biotechnology Associates, Birmingham, Ala.) in a 1:4000 dilution in blocking buffer, as previously described [2, 13].

### Statistical analysis

Data are presented as mean and *SD*, and comparison among multiple groups was performed by an ANOVA test. Comparisons of footpad swelling between *T. procumbens* and *A. sativum* groups and saline were performed with Student’s *t*-test.

## Results

### Toxicity assay

In the acute oral toxicity study of *T. procumbens* methanol and *A. sativum* aqueous extracts, none of the animals died during the assay or showed signs and symptoms of toxicity up to a dose of 2000 mg/kg. These results indicated that the extracts have a high margin of safety. The loss of body weight as a toxicity indicator was within the normal parameters of the growth curve of BALB/c mice at day 14. Mice gained the same weight and there were no significant differences (*p* < 0.05) in weight gain (weight at the 14th day minus weight at the 1st day) in the comparison between the control group (2.6 ± 1.1) (mean ± *SD*) and the mice treated either with *T. procumbens* methanol extract (5 ± 2.5) or *A. sativum* aqueous extract (3 ± 0.70). All groups registered an equal amount of food consumed (data not shown). The weight of livers and spleens was similar among the control and experimental groups (*p* > 0.05) (data not shown), and no macroscopic signs of damage were observed in these organs for either extract.

GOT and GPT levels were measured and the data showed no significant differences in transaminases (*p* > 0.05) between the control group and the group treated with *T. procumbens* or *A. sativum* (Table 1).

**Table 1.** Transaminase levels of GOT and GPT (UI) in mice treated with *T. procumbens* methanol, *A. sativum* aqueous or saline solution.

Extracts	TGO	TGP
Control	308.6 ± 168.9	58.9 ± 18.0
*T. procumbens*	346.1 ± 228.3	47.7 ± 15.8
*A. sativum*	289.7 ± 152.6	62. 6± 13.0

Each result is mean ± *SD* of TGO and TGP (UI) for three or six mice samples.

### Effect of *T. procumbens, Allium sativum,* and mixed extracts on *L. mexicana in vivo*

**Figure 1.**
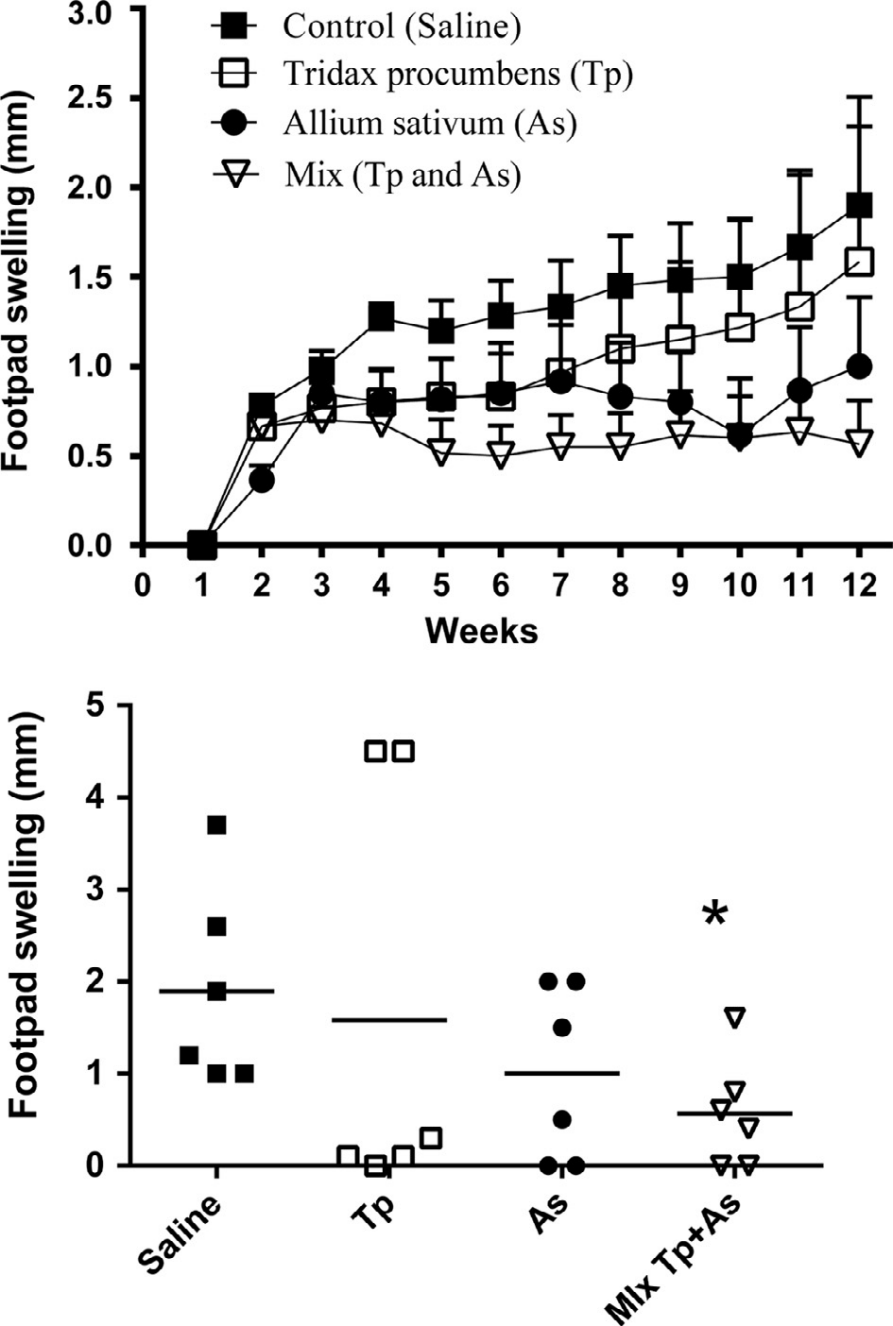
Footpad swelling in CD-1 mice after two weeks of infection with 1 × 10^6^ *L. mexicana* promastigotes. Top: four groups of mice were treated either with saline, *T. procumbens* extract, *A. sativum* extract or the mixture of *T. procumbens* and *A. sativum* extracts. The treatments were applied i.p. daily for two weeks. Bottom: the footpad thickness was measured and compared to the noninoculated footpad for each mouse for 12 weeks. Each point represents the average increase of the infected footpad thickness, standard error (*n* = 6). Comparison of four experimental groups in 12 weeks after infection, each symbol represents each mouse of the group, the horizontal line represents the mean, and * indicates significant differences with the PBS-treatment control group (unpaired *t*-test, *p* < 0.05).

The infection with *L. mexicana* induced a progressive increase in the size of the footpad of CD-1 mice. Both *T. procumbens* methanol extract and *A. sativum* aqueous extract in separate treatments showed a tendency to reduce the development of the cutaneous lesion in mice; however, four out of six mice treated with *T. procumbens* methanol extract presented minor lesions, while the other two did not respond to the treatment and developed severe lesions (Fig. 1 bottom). This variability can be explained by the genetic diversity of the CD-1 strain. The mixture of both extracts showed the best activity, significantly reducing the development of the lesion caused by *L. mexicana* (Fig. 1).

**Figure 2.**
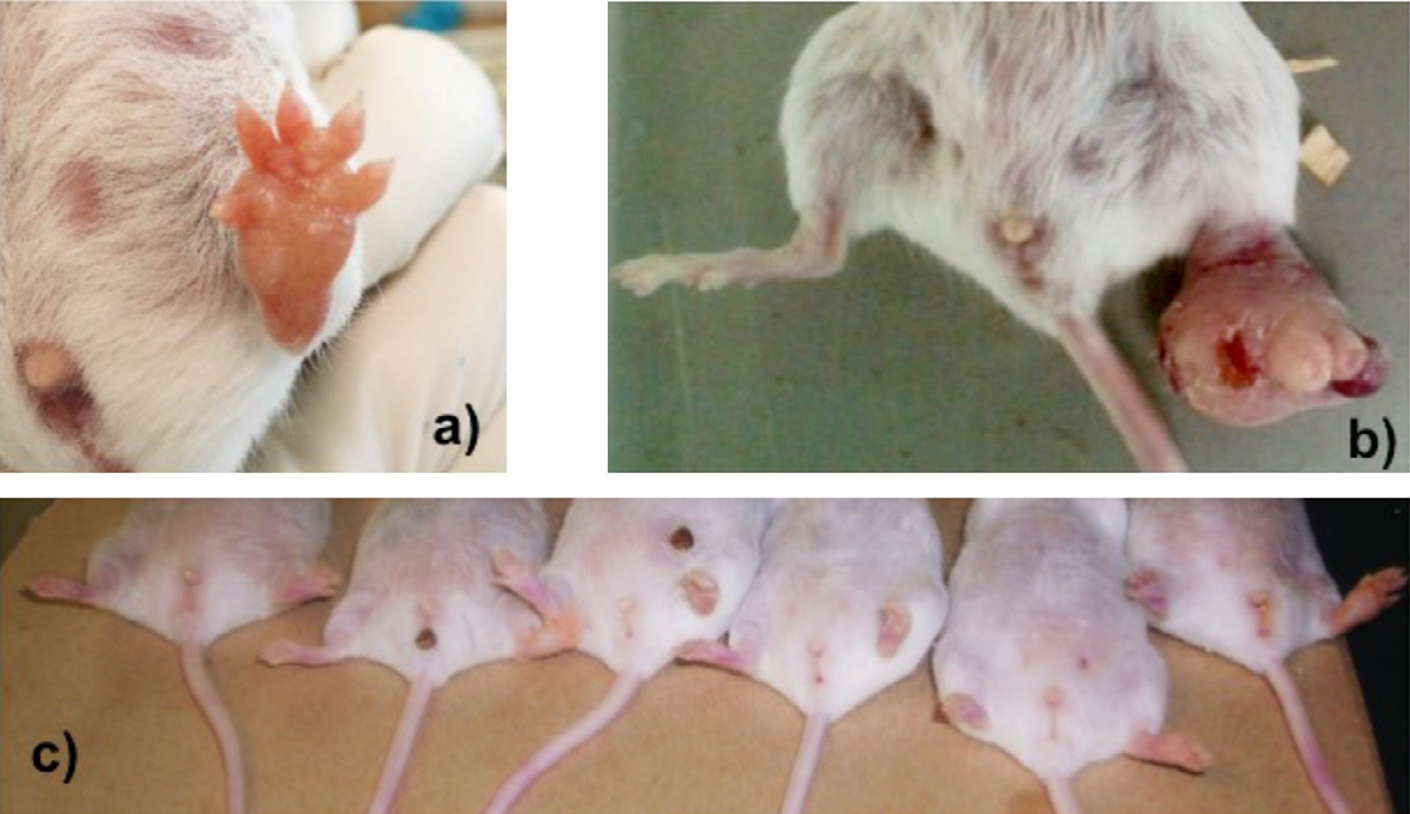
Appearance of the lesions caused by *Leishmania mexicana* with and without the treatment (mixture of *T. procumbens* + *A. sativum* extracts) 12 weeks after infection. (a), (b) Deformed and ulcerated lesions on left foot of untreated mice. (c) Controlled and healed lesions on left foot of mice treated with the mixture.

In general, lesions observed in mice treated with the mixture of *T. procumbens* and *A. sativum* did not present necrosis or cause damage to the skin compared with the control group treated with saline solution after day 12 (Fig. 2).

**Figure 3.**
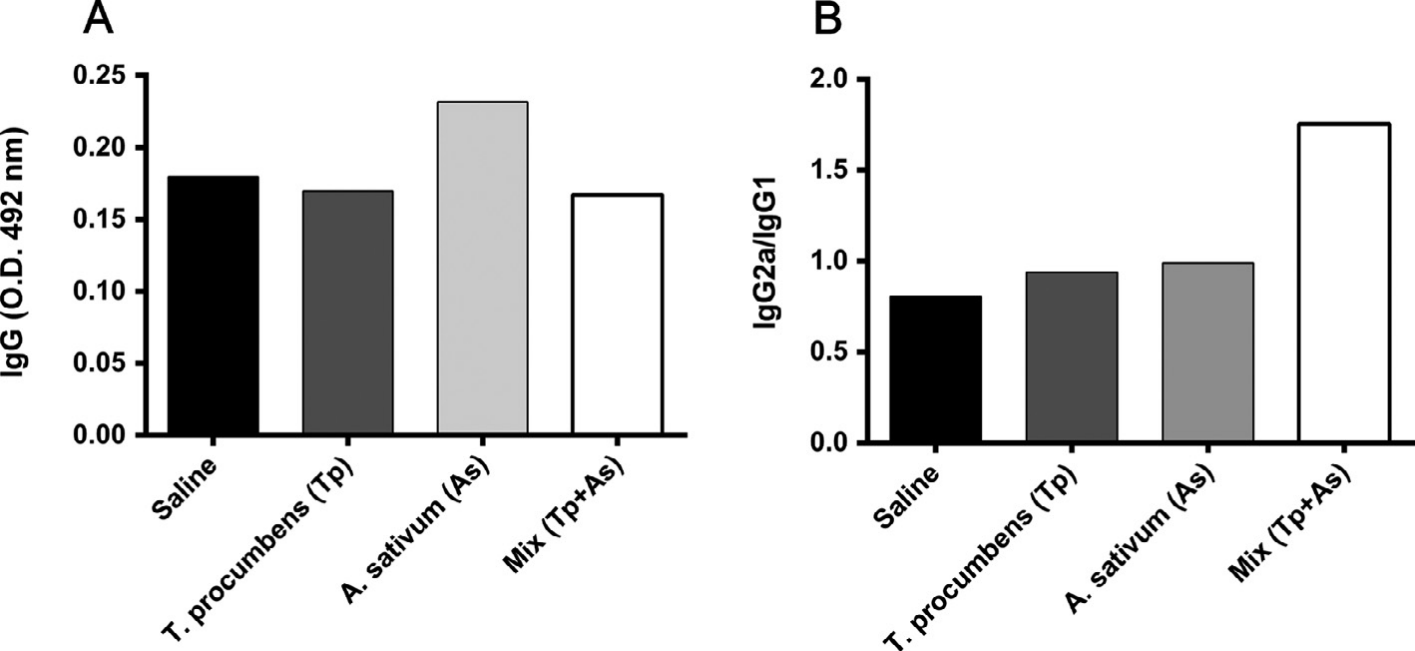
Total levels of IgGs, ratio IgG2a/IgG1 in mice with different treatments compared with PBS-treatment control group. Total IgGs relationship with general humoral immune response and IgG2a/IgG1 related to Th1-type immune response causing protection of cutaneous leishmaniasis. Control group (black bar), *T. procumbens* group (deep gray bar), *A. sativum* group (light gray bar) and Mix group (white bar).

In order to understand the function of the mixture in humoral immune responses to control the infection, we measured the level of total IgG, IgG1, and IgG2a against *L. mexicana* in serum of mice treated either with *T. procumbens*, *A. sativum*, or the mixture of both compared with mice without treatment (Fig. 3). The means of antibody levels of each group were compared. Previous studies have demonstrated that the immunomodulatory effect of *A. sativum* extract is due to an increase in IFN-*γ* and NO in infected mice [12]. In the present study it was shown that *A. sativum* extract is also able to promote the production of antibodies (Fig. 3A). The group treated with *A. sativum* showed the highest total IgG production compared with the control group; however, the highest ratio of IgG2a/IgG1 was observed in the mixture group (Fig. 3B).

## Discussion

The results of the toxicity assay showed that the limit dose of 2000 mg/kg was innocuous to mice, and they were consistent with another toxicity study which demonstrated that *T. procumbens* methanol extract was not toxic at 5 g/kg body weight in rats, confirming a high margin of safety [23]. According to these results, *T*. *procumbens* LD_50_ is located over 2000 mg/kg body weight. Regarding the toxicity of *A. sativum* extract, previous studies have revealed its curative and protective effects in a nephrotoxicity model [27] and a protective effect in the kidney and liver of rats [17]. Accordingly, the present study shows no toxicity of *A. sativum* aqueous extract in livers of mice receiving an oral dose of 2000 mg/kg.

The simultaneous application of the mixed extract (*T. procumbens* and *A. sativum* extracts) avoided the development of a lesion; to our knowledge, there are no other studies reported using a mixture of two bioactive plant extracts against *Leishmania* spp. In a previous study, we demonstrated that *A. sativum* aqueous extract had a visible effect on the reduction of the lesion caused by *L. mexicana* [12]. This was confirmed by Wabwoba et al., who also concluded that the mechanism of action of *A. sativum* is apparently immunomodulatory, and that *A. sativum* compounds should be purified and tried as complementary medicine in the management of leishmaniasis [32]. However, we believe that these components perhaps do not need to be purified to obtain a good treatment, they just have to be standardized [14, 21].

The highest IgG2a/IgG1 ratio in mice treated with mixed extract, which controlled the progress of infection for *L. mexicana*, suggests that the simultaneous application of *T. procumbens* and *A. sativum* extracts redirects the immune response caused by the infection to a Th1-type immune response. This explains why the observed lesions in this group are less than those of the other experimental groups, as this type of response is considered most suitable for the control of infection [2].

There are reports on *T. procumbens* aqueous extract inducing an immunomodulatory response in mice [30] and immunomodulatory activity in *T. procumbens* methanol extract has been detected [1]. Phytochemical studies performed on *T. procumbens* against *L. mexicana* led to the isolation of an oxylipin, highly selective against promastigotes of *L. mexicana* [20]. This metabolite was tested in vitro against intracellular amastigotes, showing an IC_50_ = 0.48 μg/mL [19]. This suggests that *T. procumbens* methanol extract could have a direct effect not only on the amastigotes, but also an additive effect with *A. sativum* in modulating the immune response, so that the simultaneous application of both extracts has a more efficient therapeutic effect. Similar results were obtained in a study which demonstrated an in vivo immunomodulatory effect with direct and indirect actions with a flower extract of *Tilia* sp. to improve the antitumoral activity [9].

Our work suggests that *T. procumbens* and *A. sativum* extracts used together control localized cutaneous leishmaniasis caused by *L. mexicana*.
